# Awareness, risk perception, and attitudes toward prostate cancer detection and MRI among Armenian men

**DOI:** 10.3389/fpubh.2026.1868400

**Published:** 2026-07-15

**Authors:** N. N. Shalunc, S. S. Gaboyan, N. G. Matinyan, T. S. Mnatsakanyan, M. G. Hovhannisyan, I. J. Ergas

**Affiliations:** 1 Faculty of Public Health, Yerevan State Medical University Named After Mkhitar Heratsi, Yerevan, Armenia; 2 Proton Diagnostic and Educational Center, Yerevan, Armenia; 3 Department of Surgery, University of California San Diego, La Jolla, CA, United States; 4 Division of Research, Kaiser Permanente Northern California, Pleasanton, CA, United States

**Keywords:** Armenia, awareness, early detection, magnetic resonance imaging, prostate cancer, PSA, risk perception

## Abstract

**Background:**

Prostate cancer is a common malignancy and major cause of cancer mortality among men worldwide. Early detection improves outcomes, but participation depends on awareness, perceived risk, and attitudes toward testing and diagnostic pathways. In Armenia, many cases are diagnosed at advanced stages, partly due to limited screening practices and low public awareness. This study assessed Armenian men’s knowledge of prostate cancer, perception of personal risk, and attitudes toward prostate MRI within early-detection pathways, including perceived barriers to undergoing MRI.

**Methods:**

A cross-sectional study was conducted from December 2024 to March 2025 at the Proton Diagnostic and Educational Center in Yerevan, Armenia. The study included 196 men aged ≥45 years undergoing MRI examinations unrelated to prostate conditions. Participants completed a structured 38-item questionnaire adapted from validated international surveys assessing awareness, perceived risk, screening attitudes, and barriers to MRI screening. Four perception scores were derived from Likert-scale responses. Associations between sociodemographic factors and perception scores were examined using multivariable linear regression analysis.

**Results:**

The mean perceived risk score was 2.95 (SD = 0.73) and the mean awareness score was 3.21 (SD = 0.72). Men aged 56–65 years had a 0.30-point higher perceived risk than those aged 45–55 years (mean difference. = 0.30; 95% CI: 0.05–0.55). Awareness was 0.32 points lower among men with secondary education or less (mean difference = −0.32; 95% CI: −0.57 to −0.07). The mean screening confidence score was 3.45 (SD = 0.81). Perceived barriers to MRI screening were higher among men with secondary education or less (mean difference = 0.39; 95% CI: 0.18–0.60) and those with vocational or technical education (adjusted mean difference = 0.40; 95% CI: 0.19–0.61) than among those with higher education.

**Conclusion:**

Awareness of prostate cancer and early detection among Armenian men moderate and varied by education level. Lower educational attainment was consistently associated with lower awareness and greater perceived barriers to MRI-based diagnostic evaluation. Targeted health education and more equitable access to diagnostic services may support earlier detection and help reduce the burden of prostate cancer in Armenia.

## Introduction

Prostate cancer is the second most frequently diagnosed cancer in men and the fifth leading cause of cancer-related death worldwide (1–5). According to GLOBOCAN 2022, approximately 1.47 million new cases were recorded, representing 7.3% of all male cancers (6). With aging populations and changing lifestyles, global incidence is projected to nearly double by 2040, reaching about 3 million new cases annually (7). Both hereditary and lifestyle factors contribute to prostate cancer risk; systematic reviews identify genetic predisposition, obesity, and related behaviors as major influences (8, 9). Despite progress in diagnosis and therapy, the disease still causes roughly 375,000 deaths each year (4–7), with mortality highest in settings lacking early detection and treatment (3, 10).

In Armenia, prostate cancer ranks among the most common cancers in men. National statistics recorded 568 new cases in 2024, second only to lung cancer (11). Mortality remains substantial, in part because many patients are diagnosed at advanced stages (i.e., locally advanced or metastatic disease, characterized by tumor extension beyond the prostate and/or distant metastases). Contributing factors include the absence of national screening programs, limited diagnostic infrastructure, and low public awareness of risk factors and early symptoms (12, 13). Similar challenges in prostate cancer burden and late detection have been reported across Central and Eastern Europe, where incidence trends and outcomes reflect gaps in organized early-detection pathways (14).

Early detection is critical for improving outcomes. Prostate-specific antigen (PSA) testing has long been the primary screening tool, yet its limited specificity can result in false positives and unnecessary biopsies (15–17). Multiparametric magnetic resonance imaging (mpMRI) now serves as an important complementary diagnostic method, with randomized trials showing that mpMRI prior to biopsy improves detection of clinically significant cancer and reduces unnecessary transrectal ultrasound (TRUS) biopsies (18–21). The Prostate Imaging-Reporting and Data System (PI-RADS) has standardized MRI interpretation, but MRI remains costly, time-consuming, and less accessible than PSA testing, limiting its role as a first-line population-level approach. Given resource constraints, simplified protocols such as biparametric MRI have been proposed to improve feasibility in selected diagnostic pathways (20). Overall, these considerations support positioning prostate MRI within risk-stratified pathways—particularly for men with elevated PSA or higher baseline risk (17, 19–21).

Men’s knowledge and attitudes strongly influence participation in screening. Evidence from other countries shows that decisions about PSA testing or MRI depend on perceptions of test usefulness, trust in healthcare providers, costs, and fear of diagnosis (22–27). In many low- and middle-income (LMIC) settings, financial and cultural barriers further reduce participation (28, 29). Studies from Turkey and Lebanon report generally low to moderate awareness, shaped by education and place of residence (22, 23). In Eastern Europe and other post-Soviet contexts, late-stage diagnosis remains common due to weak health communication and lack of organized screening (6, 30, 31). Evidence from other post-Soviet settings also highlights the role of psychological and informational barriers in cancer screening participation, reinforcing the need to address both knowledge and perceptions in early-detection strategies (32).

In this context, evidence on men’s knowledge, perceptions, and attitudes toward prostate cancer and diagnostic tools remains scarce in Armenia and the broader Caucasus region. This gap limits the development of effective awareness campaigns and informed resource allocation. We hypothesized that awareness of prostate cancer and perceived personal risk would be moderate and would vary according to key sociodemographic characteristics, including age, education, and marital status. The present study therefore aimed to assess Armenian men’s awareness of prostate cancer, perception of personal risk, and attitudes toward MRI within early detection pathways. Findings are expected to inform national health policy, guide educational strategies, and support more accessible, cost-effective approaches to early detection.

## Materials and methods

### Study design and setting

This quantitative, cross-sectional study was conducted at the Proton Diagnostic and Educational Center in Yerevan, Armenia, from December 2024 to March 2025. The Center is a tertiary-level facility equipped with advanced imaging technologies, including a 3-Tesla MRI scanner, and serves both Yerevan and regional populations. The site was selected because of its central role in MRI diagnostics and its ability to recruit participants from diverse sociodemographic backgrounds.

### Study population and sampling

The study population included men aged ≥45 years who underwent diagnostic MRI examinations during the study period. Eligible participants were scheduled for any type of MRI except those referred specifically for prostate or pelvic imaging. Pelvic imaging referrals were excluded because these examinations may increase awareness of genitourinary conditions and potentially influence responses. Men with a known or suspected diagnosis of prostate cancer were excluded to reduce bias related to prior awareness or personal experience. Men younger than 45 years were excluded because screening and early-detection efforts typically target middle-aged and older men.

Participants were recruited using consecutive sampling. All eligible men attending the Center during the data-collection period were invited to participate, and recruitment continued until the target sample size was reached. Participation was voluntary, and written informed consent was obtained prior to questionnaire administration.

The study protocol was approved by the Ethics Committee of Yerevan State Medical University after Mkhitar Heratsi (Protocol No. N12-11/24; approval date: 13 June 2024).

### Sample size

The sample size was calculated using the single-proportion formula n=Z2pq/d2.

with Z = 1.96 (95% confidence level), *p* = 0.5, q = 0.5, and d = 0.07 (margin of error, a 7% margin of error was selected to balance statistical precision with study feasibility, given the exploratory nature of the study and the absence of prior national data on prostate cancer awareness among Armenian men). This yielded a required sample of 196 participants.

### Data collection tool

Data were collected using a structured 38-item questionnaire adapted from previously validated international instruments assessing prostate cancer awareness, perceived risk, and attitudes toward early detection (22, 33). The questionnaire included four domains: (1) sociodemographic characteristics (6 items: age, marital status, education, self-rated health, region of residence, and place of residence (urban/rural)); (2) perceived risk (8 items assessing lifetime, 10-year, and 5-year risk; comparative risk relative to peers with genetic predisposition or obesity; perceived mortality risk; and beliefs about age and family history as determinants), measured on a five-point Likert scale; (3) awareness of symptoms (5 items assessing recognition of reduced urine output, frequent urination, burning/itching during urination, hematuria, and pain during urination); and (4) awareness, attitudes, and barriers (19 items), including general awareness of the prostate, prostate cancer, and PSA testing (yes/no), attitudes toward early detection (five-point Likert scale), and perceived barriers to MRI (11 items; five-point Likert scale) such as cost, lack of symptoms, lack of family history, fear, distrust, and concerns about treatment.

The questionnaire was translated into Armenian and reviewed for linguistic and cultural appropriateness by the research team and subject-matter experts. A pilot test was conducted with 15 men from the target population to assess clarity, comprehension, and cultural relevance, and minor linguistic revisions were made accordingly. The final questionnaire was self-administered and took approximately 10–15 min to complete; research staff provided assistance when clarification was needed. Although the adapted questionnaire demonstrated acceptable internal consistency (Cronbach’s alpha > 0.75), additional psychometric analyses such as exploratory factor analysis, confirmatory factor analysis, and construct validity assessment were not performed. This was because the objective of the study was to use an adapted instrument to assess awareness, risk perception, and attitudes within the study population rather than to develop or comprehensively validate a new measurement scale.

### Perception scores

Four composite scores were calculated as the mean of item responses within each domain: perceived risk (8 items), awareness (5 items), screening confidence (5 items), and MRI barriers (11 items). Items included in these scores were coded on a 1-to-5 scale, with higher scores indicating greater perceived risk, greater awareness, greater confidence in early detection, and greater perceived barriers to MRI screening. Because response options were not numerically coded on the questionnaire, values were assigned during analysis to ensure that higher scores consistently reflected greater levels of each construct. Binary yes/no awareness items were not included in the composite scores. Questions number 6 and 8 under section 2 of the questionnaire were rescaled due to less answer choices. Internal consistency was acceptable for all four scales (Cronbach’s alpha>0.75). For descriptive purposes, scores were converted to percentages of the maximum attainable score and categorized as low (<50%), moderate (50–75%), and high (>75%).

### Statistical analysis

Cleaning procedures included range and consistency checks. Descriptive statistics were computed for all variables, with categorical data summarized as frequencies and percentages and continuous data as mean and standard deviation (SD). Associations between sociodemographic variables (age, education, residence, marital status, and self-rated health) and perception scores were examined using univariate and multivariable linear regression models to estimate unadjusted and adjusted mean differences with 95% confidence intervals (CI). Underlying assumptions of linear regression were assessed, including residual normality, homoscedasticity, and the absence of influential observations. No substantial violations were identified. Statistical significance was defined as *p* < 0.05 (two-sided).

All data were entered using SPSS (version 21, IBM Corp.) and analyzed using Stata (version 19.5, StataCorp, College Station, TX).

## Results

### Participants’ characteristics

A total of 196 men participated in the study (Table 1). Nearly half (48.5%) were aged 45–55 years, while 25.5% were aged 66 years or older. Most participants were married (83.2%) and reported at least secondary education, with 49.5% holding higher education degrees. The majority (74.5%) resided in urban areas, including 41.3% in Yerevan. Regarding self-rated health, 37.2% described their health as very good or excellent, 37.8% as good, 23.0% as fair, and 2.0% as poor.

**Table 1:** Sociodemographic characteristics of study participants (*N* = 196).

Continuous variables	Mean (sd)
Perceived risk score	2.95 (0.73)
Awareness score	3.21 (0.72)
Screening confidence score	3.45 (0.81)
Barriers score	2.40 (0.61)
Categorical variables, n (%)	
Age, years	
45–55	95 (48.5%)
56–65	51 (26.0%)
> 65	50 (25.5%)
Marital status	
Married	163 (83.2%)
Divorced	16 (8.2%)
Single	17 (8.7%)
Education level	
Higher education	97 (49.5%)
Secondary education or less	52 (26.5%)
Vocational or technical	47 (24.0%)
Perceived health	
Excellent	34 (17.3%)
Very good	39 (19.9%)
Good	74 (37.8%)
Fair	45 (23.0%)
Poor	4 (2.0%)
Region	
Yerevan	81 (41.3%)
Other Urban	65 (33.2%)
Rural	50 (25.5%)

Item-level analysis (Table 2) showed relatively high recognition of the prostate and prostate cancer as concepts, but substantially lower awareness of PSA testing and only recognition of specific symptoms. Overall, 85.2% of participants reported having heard of prostate cancer, and 89.3% correctly identified the prostate. Awareness of the PSA test was substantially lower, with only 42.9% reporting familiarity with it. Awareness of individual symptoms was moderate and varied by symptom type. Pain during urination (57.7%) and hematuria (54.5%) were the most commonly recognized symptoms, followed by decreased urine output or difficulty urinating (50.5%). Lower recognition was observed for frequent urination (44.9%) and burning or itching during urination (42.3%). These findings suggest that while general awareness of prostate cancer was relatively high, knowledge of specific symptoms and early detection tools remained less consistent.

**Table 2:** Item-level awareness of prostate cancer, PSA, and selected symptoms among study participants (*N* = 196).

Item	Yes / Agree n (%)	Neutral n (%)	No / Disagree n (%)
Heard of prostate cancer	167 (85.2)	—	29 (14.8)
Aware of what the prostate is	175 (89.3)	—	21 (10.7)
Aware of the PSA test	84 (42.9)	—	112 (57.1)
Decreased urine output / difficulty urinating	99 (50.5)	46 (23.5)	51 (26.0)
Frequent urination	88 (44.9)	38 (19.4)	70 (35.8)
Burning and itching during urination	83 (42.3)	52 (26.5)	61 (31.2)
Presence of blood in urine	107 (54.5)	41 (20.9)	48 (24.5)
Pain during urination	113 (57.7)	35 (17.9)	48 (24.5)

### Perceived risk of prostate cancer score

The mean perceived risk score was 2.95 (SD = 0.73). In multivariable analysis (Table 3), after adjustment for age, marital status, education level, self-rated health, and residence, men aged 56–65 years had a 0.30-point higher perceived risk score than men aged 45–55 years (mean difference = 0.30; 95% CI: 0.05–0.55; *p* < 0.05). In addition, men who rated their health as fair had a 0.43-point higher perceived risk score (mean difference = 0.43; 95% CI: 0.11–0.76; *p* < 0.01), while those who rated their health as poor had an even higher perceived risk score (mean difference = 0.82; 95% CI: 0.06–1.58; *p* < 0.05) compared with men who rated their health as excellent.

**Table 3:** Factors associated with perceived risk and awareness scores in unadjusted and adjusted linear regression analyses (*N* = 196).

Variables	Unadjusted			Adjusted^a^		
	Mean difference	95% CI	*p*-value	Mean difference	95% CI	*p*-value
Age						
45–55 years	Ref.	–	–	Ref.	–	–
56–65 years	0.30	0.05–0.55	0.018*	0.29	0.03–0.54	0.026*
66 years and older	0.22	−0.02–0.47	0.076	0.22	−0.04–0.47	0.092
Marital status						
Married	Ref.	–	–	Ref.	–	–
Divorced	0.09	−0.28–0.47	0.623	0.08	−0.29–0.46	0.671
Single	−0.20	−0.57–0.17	0.286	−0.11	−0.49–0.27	0.564
Education level						
Higher education	Ref.	–	–	Ref.	–	–
Secondary education or less	−0.13	−0.38–0.11	0.288	−0.15	−0.41–0.11	0.261
Vocational or technical	−0.20	−0.45–0.06	0.131	−0.24	−0.50–0.02	0.072
Perceived health						
Excellent	Ref.	–	–	Ref.	–	–
Very good	0.27	−0.06–0.60	0.113	0.21	−0.12–0.55	0.213
Good	0.35	0.05–0.64	0.021*	0.29	−0.01–0.59	0.051
Fair	0.48	0.16–0.80	0.00**4	0.43	0.11–0.76	0.009**
Poor	0.86	0.11–1.60	0.024*	0.82	0.06–1.58	0.036*
Region						
Yerevan	Ref.	–	–	Ref.	–	–
Other Urban	−0.06	−0.30–0.18	0.620	−0.11	−0.36–0.13	0.352
Rural	−0.17	−0.43–0.09	0.188	−0.18	−0.45–0.09	0.187

CI, confidence interval; ref., reference. *p < 0.05, **p < 0.01, ***p < 0.001.

a Adjusted mean differences were estimated from multivariable linear regression models including all listed variables simultaneously; each estimate is adjusted for the other variables in the model.

### Prostate cancer symptom awareness score

The mean awareness score was 3.21 (SD = 0.72). As shown in Table 4, after adjustment for age, marital status, education level, self-rated health, and residence, men aged ≥66 years had a 0.29-point higher awareness score than men aged 45–55 years (mean difference = 0.29; 95% CI: 0.04–0.54; *p* < 0.05). Moreover, single men had a 0.43-point higher awareness score than married men (mean difference = 0.43; 95% CI: 0.06–0.80; *p* < 0.05). Men with secondary education or less had a 0.32-point lower awareness score than those with higher education (mean difference = −0.32; 95% CI: −0.57–−0.07; *p* < 0.05).

**Table 4:** Factors associated with awareness of prostate cancer score in unadjusted and adjusted linear regression analyses (*N* = 196).

Variables	Unadjusted			Adjusted^a^		
	Mean difference	95% CI	*p*-value	Mean difference	95% CI	*p*-value
Age						
45–55 years	Ref.	–	–	Ref.	–	–
56–65 years	0.13	−0.11–0.37	0.274	0.10	−0.15–0.34	0.446
66 years and older	0.37	0.13–0.62	0.003**	0.29	0.04–0.54	0.023*
Marital status						
Married	Ref.	–	–	Ref.	–	–
Divorced	0.10	−0.27–0.46	0.602	0.07	−0.29–0.44	0.693
Single	0.45	0.09–0.80	0.014*	0.43	0.06–0.80	0.023*
Education level						
Higher education	Ref.	–	–	Ref.	–	–
Secondary education or less	−0.24	−0.48–0.01	0.053	−0.32	−0.57–−0.07	0.013*
Vocational or technical	−0.13	−0.38–0.12	0.295	−0.25	−0.50–0.01	0.059
Perceived health						
Excellent	Ref.	–	–	Ref.	–	–
Very good	−0.08	−0.41–0.25	0.650	−0.03	−0.35–0.30	0.868
Good	0.08	−0.21–0.38	0.566	0.10	−0.19–0.39	0.485
Fair	0.13	−0.19–0.45	0.434	0.17	−0.14–0.49	0.281
Poor	0.65	−0.10–1.39	0.088	0.60	−0.14–1.34	0.113
Region						
Yerevan	Ref.	–	–	Ref.	–	–
Other Urban	0.19	−0.04–0.43	0.105	0.12	−0.12–0.35	0.330
Rural	0.10	−0.16–0.35	0.453	0.10	−0.16–0.37	0.437

CI, confidence interval; ref., reference. *p < 0.05, **p < 0.01, ***p < 0.001.

a Adjusted mean differences were estimated from multivariable linear regression models including all listed variables simultaneously; each estimate is adjusted for the other variables in the model.

### Screening confidence score

The mean screening confidence score was 3.45 (SD = 0.81). In multivariable analysis (Table 5), after adjustment for age, marital status, education level, self-rated health, and residence, men with vocational or technical education had a 0.52-point lower screening confidence score than those with higher education (mean difference = −0.52; 95% CI: −0.81–−0.23; *p* < 0.01).

**Table 5:** Factors associated with screening confidence score in unadjusted and adjusted linear regression analyses (*N* = 196).

Variables	Unadjusted			Adjusted^a^		
	Mean difference	95% CI	*p*-value	Mean difference	95% CI	*p*-value
Age						
45–55 years	Ref.	–	–	Ref.	–	–
56–65 years	0.21	−0.07–0.48	0.136	0.24	−0.04–0.52	0.097
66 years and older	−0.06	−0.34–0.22	0.676	0.01	−0.27–0.29	0.959
Marital status						
Married	Ref.	–	–	Ref.	–	–
Divorced	−0.22	−0.64–0.19	0.296	−0.24	−0.65–0.18	0.265
Single	−0.27	−0.68–0.13	0.186	−0.04	0.46–0.38	0.847
Education level						
Higher education	Ref.	–	–	Ref.	–	–
Secondary education or less	−0.23	−0.50–0.03	0.086	−0.20	−0.48–0.09	0.181
Vocational or technical	−0.52	−0.79–−0.24	<0.001***	−0.52	−0.81–−0.23	0.001**
Perceived health						
Excellent	Ref.	–	–	Ref.	–	–
Very good	−0.19	−0.56–0.18	0.316	−0.23	−0.60–0.14	0.226
Good	−0.11	−0.44–0.22	0.501	−0.18	−0.51–0.14	0.267
Fair	0.08	−0.29–0.44	0.677	0.06	−0.30–0.42	0.735
Poor	−0.32	−1.17–0.52	0.449	−0.10	−0.95–0.74	0.809
Region						
Yerevan	Ref.	–	–	Ref.	–	–
Other Urban	0.09	−0.18–0.35	0.511	−0.03	−0.30–0.24	0.825
Rural	−0.09	−0.37–0.20	0.550	−0.15	−0.45–0.14	0.310

CI, confidence interval; ref., reference. *p < 0.05, **p < 0.01, ***p < 0.001.

a Adjusted mean differences were estimated from multivariable linear regression models including all listed variables simultaneously; each estimate is adjusted for the other variables in the model.

### Perceived barriers to prostate MRI score

The mean MRI screening barriers score was 2.40 (SD = 0.61). As shown in Table 6, after adjustment for age, marital status, education level, self-rated health, and residence, men with secondary education or less had a 0.39-point higher MRI screening barriers score than men with higher education (mean difference = 0.39; 95% CI: 0.18–0.60; *p* < 0.001) and those with vocational or technical education had a 0.40-point higher score (mean difference = 0.40; 95% CI: 0.19–0.61; *p* < 0.001). Furthermore, men who rated their health as fair had 0.28-point lower MRI screening barriers score than men who rated their health as excellent (mean difference = −0.28; 95% CI: −0.54–−0.02; *p* < 0.05).

**Table 6:** Factors associated with MRI screening barriers score in unadjusted and adjusted linear regression analyses (*N* = 196).

Variables	Unadjusted			Adjusted^a^		
	Mean difference	95% CI	*p*-value	Mean Difference	95% CI	*p*-value
Age						
45–55 years	ref.	–	–	ref.	–	–
56–65 years	−0.24	−0.44–−0.03	0.022	−0.19	−0.39–0.01	0.065
66 years and older	−0.17	−0.38–0.04	0.106	−0.18	−0.38–0.03	0.086
Marital status						
Married	ref.	–	–	ref.	–	–
Divorced	0.30	−0.01–0.61	0.055	0.26	−0.05–0.56	0.095
Single	0.15	−0.16–0.45	0.340	0.03	−0.27–0.34	0.823
Education level						
Higher education	ref.	–	–	ref.	–	–
Secondary education or less	0.34	0.14–0.54	0.001**	0.39	0.18–0.60	<0.001***
Vocational or technical	0.35	0.14–0.55	0.001**	0.40	0.19–0.61	<0.001***
Perceived health						
Excellent	ref.	–	–	ref.	–	–
Very good	−0.18	−0.46–0.10	0.203	−0.17	−0.44–0.10	0.224
Good	−0.21	−0.45–0.04	0.098	−0.19	−0.43–0.05	0.115
Fair	−0.28	−0.55–−0.01	0.046*	−0.28	−0.54–−0.02	0.038*
Poor	−0.43	−1.06–0.20	0.177	−0.56	−1.17–0.05	0.074
Region						
Yerevan	ref.	–	–	ref.	–	–
Other Urban	−0.10	−0.30–0.10	0.314	−0.02	−0.21–0.18	0.852
Rural	−0.09	−0.31–0.12	0.408	−0.13	−0.35–0.08	0.233

CI, confidence interval; ref., reference. *p < 0.05, **p < 0.01, ***p < 0.001.

a Adjusted mean differences were estimated from multivariable linear regression models including all listed variables simultaneously; each estimate is adjusted for the other variables in the model.

## Discussion

This study provides rare and timely quantitative evidence from Armenia on men’s awareness of prostate cancer, perceived personal risk, screening confidence, and perceived barriers to prostate MRI. Overall, awareness and screening confidence were moderate, but important disparities were observed across some sociodemographic groups. Educational level emerged as the most consistent factor associated with awareness, screening confidence, and MRI-related barriers. These findings are particularly important in Armenia, where prostate cancer is a common malignancy among men and late-stage diagnosis remains frequent (6, 11, 13). Similar challenges in early detection and delayed diagnosis have been reported across Central and Eastern Europe, highlighting the potential for comparable structural barriers in the region (14).

A key finding of this study was the consistent pattern of disadvantage observed among men with lower educational attainment. Participants with secondary education or less had lower awareness scores, while those with secondary or vocational education reported greater MRI barriers. Men with vocational or technical education also demonstrated lower screening confidence. These findings are consistent with evidence from other settings showing that educational level influences health literacy, cancer awareness, understanding of risk, and engagement with preventive health services (23–25, 27, 29). In settings without organized screening programs, such inequalities may be more pronounced, as men rely more heavily on individual knowledge, initiative, and communication with healthcare providers to access early detection (24, 25, 27, 28).

Age-related differences were also observed. Men aged 56–65 years reported higher perceived risk than those aged 45–55 years, while men aged 66 years and older demonstrated higher awareness. These patterns may reflect increasing exposure to health information, more frequent contact with healthcare providers, and greater recognition of cancer risk with advancing age (23, 25, 27). At the same time, the findings suggest that younger men within the age range relevant to early detection may underestimate their vulnerability. This is important because perceived risk is a key determinant of preventive behavior and testing uptake (25–27). If men in their late 40s and early 50s do not view prostate cancer as a relevant health concern, opportunities for timely counseling and earlier diagnostic evaluation may be reduced (15, 27).

Self-rated health was also associated with perception scores. Men who rated their health as fair or poor had higher perceived risk scores than those reporting excellent health, suggesting that subjective health status may influence perceived vulnerability to cancer. In addition, men with fair self-rated health reported lower MRI barrier scores than those with excellent health. One possible explanation is that individuals who perceive themselves as less healthy may be more open to diagnostic evaluation, whereas those who consider themselves healthy may see less value in investigation in the absence of symptoms. This has particular relevance for prostate cancer, which may remain asymptomatic in its early stages and therefore often requires awareness-based rather than symptom-driven help-seeking (15, 17, 26, 27).

Although awareness in this sample was not very low, it remained only moderate, indicating incomplete knowledge of prostate cancer and its warning signs. This is important because awareness affects multiple stages of the pathway to diagnosis, including recognition of symptoms, interpretation of personal risk, willingness to seek medical advice, and acceptance of recommended testing (24–27). In Armenia, where there is no national screening program and public awareness efforts remain limited, such knowledge gaps may contribute to delayed diagnosis and poorer outcomes (11–13).

The mean screening confidence score was somewhat higher than the awareness score, suggesting that participants were generally favorable toward early detection. However, positive attitudes alone may not translate into behavior when informational and structural barriers remain. Men may support the idea of early detection but still be uncertain about when testing is appropriate, which diagnostic approach is most suitable, or how to access recommended services. Similar gaps between favorable attitudes and actual uptake have been described in studies of PSA testing and prostate cancer screening decisions in other countries (15, 26, 27, 31). These findings suggest that public health strategies should combine general awareness-raising with clear, practical information about when and how men should seek further evaluation (15, 24, 27).

The findings related to prostate MRI are especially relevant in the evolving diagnostic landscape of prostate cancer. Multiparametric MRI is increasingly recognized as an important component of risk-stratified and pre-biopsy diagnostic pathways because it can improve detection of clinically significant cancer and reduce unnecessary biopsies (17–19). However, MRI is resource-intensive and less accessible than PSA-based testing. Streamlined MRI approaches, such as biparametric MRI, have been discussed as a way to improve feasibility and reduce resource demands in selected diagnostic pathways (20). In the present study, MRI barriers were significantly higher among men with lower educational attainment, suggesting that advanced diagnostic procedures may be acceptable in principle but less accessible in practice. In Armenia, where imaging capacity is concentrated in tertiary settings and cost may limit utilization, prostate MRI should be considered as part of a staged diagnostic pathway rather than a population-wide screening solution (12, 17, 21). Without attention to affordability, referral access, and geographic equity, expanded reliance on MRI may unintentionally widen existing inequalities (12, 21, 31).

The finding that single men had higher awareness scores than married men was unexpected, as social support and engagement with care are often associated with preventive health behaviors (34). In many studies, marriage is associated with better preventive health behavior, likely reflecting the influence of social relationships and spousal support (35–37). In this study, however, single men demonstrated higher awareness after adjustment for other factors. Given the relatively small number of single participants, this result should be interpreted cautiously. It may reflect sample-specific differences in health information-seeking or engagement with care rather than a stable population pattern. Nevertheless, it suggests that marital status may not operate uniformly across settings and should be interpreted within local social and cultural contexts (34).

These findings have several implications for public health policy and practice in Armenia. First, educational interventions should specifically target men with lower levels of formal education, using plain language and culturally appropriate communication. Second, primary care encounters represent a key opportunity to discuss prostate cancer risk, early symptoms, and available diagnostic options, particularly in the absence of organized screening (12, 13, 15, 17, 24, 27). This is especially important in Armenia, where population-level noncommunicable disease risk factors are common and prevention-oriented counseling and risk communication remain essential (38). Third, any expansion of MRI within prostate cancer diagnostic pathways should be accompanied by measures to improve affordability and equitable access (12, 17, 21, 31). Awareness initiatives should also provide practical guidance on when men should seek consultation and what diagnostic steps may follow, rather than promoting early detection only in general terms (15, 17, 27).

This study has several strengths. It addresses an important evidence gap in Armenia and the wider Caucasus region, uses a structured questionnaire adapted from previously validated international instruments, and examines multiple dimensions of perception relevant to early detection, including perceived risk, awareness, screening confidence, and MRI-related barriers (23, 33). In addition, by recruiting men undergoing MRI for non-prostate indications, the study captured views among individuals engaged with the healthcare system but not already selected because of prostate-specific concerns.

Future studies should include larger and more geographically diverse samples to confirm these findings and improve generalizability. Qualitative research may be especially useful in clarifying how cost, trust, fear, and understanding of diagnostic procedures shape attitudes toward prostate cancer detection in Armenia (26, 27, 31, 32). Intervention studies are also needed to determine whether tailored educational materials, stronger provider counseling, or more accessible diagnostic pathways can improve awareness and support earlier detection (15, 17, 24, 27).

This study has several limitations. First, its cross-sectional design does not permit causal inference. Second, participants were recruited from a single tertiary diagnostic center in Yerevan using convenience consecutive sampling, which limits generalizability to the broader male population in Armenia. Because the sample consisted of men already attending an MRI facility, participants may have had greater healthcare access, familiarity with diagnostic services, or health-seeking behavior than men in the general population. Third, the study relied on self-reported information and may therefore be subject to recall and social desirability bias. In addition, exclusion of men with known or suspected prostate cancer reduced bias related to prior diagnosis but limits applicability to higher-risk clinical populations. Although models adjusted for key sociodemographic factors, residual confounding by unmeasured risk factors may remain. Finally, although the composite perception scores showed acceptable internal consistency, further psychometric validation of the adapted Armenian instrument would strengthen future research. Despite these limitations, this study provides the first quantitative evidence on Armenian men’s perceptions of prostate cancer and related diagnostic pathways, offering useful insights for early-detection and health-education strategies in Armenia. Consequently, the findings should not be interpreted as nationally representative estimates of awareness, risk perception, or attitudes toward prostate cancer among Armenian men.

## Conclusion

This study provides important evidence that awareness of prostate cancer and early detection among Armenian men remains incomplete and socially unequal. Men with lower educational attainment face a double disadvantage, with lower awareness and screening confidence alongside greater perceived barriers to MRI. These findings support targeted public health action to improve prostate cancer literacy, strengthen communication through primary care and community outreach, and promote more equitable diagnostic pathways. Such efforts may facilitate earlier diagnosis and help reduce the burden of prostate cancer in Armenia.

## Data Availability

The original contributions presented in the study are included in the article/supplementary material, further inquiries can be directed to the corresponding author.
